# Supplementary material for: Features of Studies on Transition Interventions for Childhood Cancer Survivors: A Scoping Review
Source: Cancers (Basel). 2024 Jan 8;16(2):272. doi: 10.3390/cancers16020272 (PMC10814357; doi:10.3390/cancers16020272)
Supplement: Supplementary file 1 [file cancers-16-00272-s001.zip › Table S1-keywords and subject headings.pdf]

Table S1-keywords and subject headings

| General Population of Interest | Cancer survivors Terms | Transition Terms |
|---|---|---|
| ‘Young adult*’ OR Youth* OR ‘Prime adult*’ OR ‘Adolescen*’ OR Teen* OR Puberty OR Pubescen* OR Prepuberty OR Prepubescen* OR Preteen* OR Juvenile* OR Child* OR ‘Child, Preschool’ OR ‘Preschool*’ OR ‘Pediatric*’ OR Paediatric* OR ‘Early childhood’ OR Infan* OR ‘Infant, Newborn’ OR Neonat* OR Newborn* | Tumor* OR Neoplas* OR Cancer* OR ‘Malignant Neoplasm*’ OR Malignanc* OR ‘Neoplasm*, Malignant’ OR ‘Cancer Survivor*’ OR ‘Survivor*, Cancer’ OR ‘Long-Term Cancer Survivor*’ OR ‘Cancer Survivor*, Long-Term’ OR ‘Long Term Cancer Survivor*’ OR ‘Cancer Survivor*, Long Term’ OR ‘Survivor*, Long-Term Cancer’ OR ‘Live with cancer*’ OR ‘Live with tumor*’ OR ‘Live with Neoplasm*’ OR ‘Live with Malignanc*’) | (transition OR ‘Health Transition’ OR ‘Continuity of Patient Care’ OR ‘Patient Care Continuity’ OR ‘Continuum of Care’ OR ‘Care Continuum’ OR ‘Continuity of Care’ OR ‘Care Continuity’ OR ‘Aftercare’ OR ‘After Care’ OR ‘After Treatment*’ OR ‘Follow-Up Care*’ OR ‘Hospital to Home Transition’ OR ‘Hospital to Home*’ OR ‘Patient discharge*’ OR ‘Discharge Planning*’ OR ‘Hando* to adult care’ OR ‘Hand OVER* to adult care’ OR ‘Hand OUT* to adult care’ OR ‘Signo* to adult care’ OR ‘Sign OVER* to adult care’ OR ‘Sign OUT* to adult care’ OR ‘Health transition*’ OR ‘Healthcare transition*’ OR ‘Health care transition*’ OR ‘Nursing hando*’ OR ‘Patient hando*’ OR ‘Patient Hand OVER*’ OR ‘Patient Hand OUT*’ OR ‘Patient Sign OUT*’ OR ‘Patient Sign OVER*’ OR ‘Patient signo*’ OR ‘Nursing Hando*’ OR ‘Nursing Hand OVER*’ OR ‘Nursing Hand OUT*’ OR ‘Clinical hando*’ OR ‘Patient transfer*’ OR ‘Transfer* to adult care’ OR ‘Transition* to adult*’ OR ‘Transition* to adult care’ OR ‘Patient Transition*’ OR ‘Care Transition*’ OR ‘Transition of Care’ OR ‘Transition of health Care’ OR ‘Health Care Transition’ OR ‘Health Care Transitions’ OR ‘Patient Turfing*’ OR ‘Patient Dumping’ OR ‘Care Retention’ OR ‘Transfer from Pediatric to Adult Care’ OR ‘Pediatric Transition To Adult Care’ OR ‘Transfer* to Adult Care’ OR ‘Transition* Care*’ OR ‘Home Transition*’) |
