# Supplementary material for: Features of Studies on Transition Interventions for Childhood Cancer Survivors: A Scoping Review
Source: Cancers (Basel). 2024 Jan 8;16(2):272. doi: 10.3390/cancers16020272 (PMC10814357; doi:10.3390/cancers16020272)
Supplement: Supplementary file 1 [file cancers-16-00272-s001.zip › Table S2-search strategy.pdf]

Table S2-search strategy in Ovid MEDLINE(R) ALL <2005 to October 17, 2022>

Number	search strategy	search results
1	young adult*.ab. or young adult*.at.	126083
2	youth*.ab. or youth*.at.	99172
3	prime adult*.ab. or prime adult*.at.	42
4	adolescen*.ab. or adolescen*.at.	353802
5	teen*.ab. or teen*.at.	35468
6	puberty.ab. or puberty.at.	33388
7	pubescen*.ab. or pubesceb*.at.	3204
8	prepuberty.ab. or prepuberty.at.	770
9	prepubescen*.ab. or prepubescen*.at.	1323
10	preteen*.ab. or preteen*.at.	460
11	juvenile*.ab. or juvenile*.at.	96005
12	child*.ab. or child*.at.	1631259
13	child, preschool.ab. or child, preschool.at.	36
14	preschool*.ab. or preschool*.at.	34456
15	pediatric*.ab. or pediatric*.at.	414672
16	paediatric*.ab. or paediatric*.at.	93072
17	early childhood.ab. or early childhood.at.	33535
18	infan*.ab. or infan*.at.	497342
19	infant, newborn.ab. or infant, newborn.at.	108
20	neonat*.ab. or neonat*.at.	331869
21	newborn*.ab. or newborn*.at.	167616
22	1 or 2 or 3 or 4 or 5 or 6 or 7 or 8 or 9 or 10 or 11 or 12 or 13 or 14 or 15 or 16 or 17 or 18 or 19 or 20 or 21	2780169
23	tumor*.ab. or tumor*.at.	1827065
24	neoplasm*.ab. or neoplasm*.at.	165761
25	neoplasia*.ab. or neoplasia*.at.	72696
26	cancer*.ab. or cancer*.at.	2157315
27	malignant neoplasm*.ab. or malignant neoplasm*.at.	15963
28	neoplasm*, malignant.ab. or neoplasm*, malignant.at.	262
29	cancer survivor*.ab. or cancer survivor*.at.	24533
30	Survivor*, Cancer.ab. or Survivor*, Cancer.at.	555
31	long-term cancer survivor*.ab. or long-term cancer survivor*.at.	537
32	cancer survivor*, long-term.ab. or cancer survivor*, long-term.at.	51
33	Long Term Cancer Survivor*.ab. or Long Term Cancer Survivor*.at.	537
34	Cancer Survivor*, Long Term.ab. or Cancer Survivor*, Long Term.at.	51
35	Survivor*, Long-Term Cancer.ab. or Survivor*, Long-Term Cancer.at.	7
36	Live with cancer*.ab. or Live with cancer*.at.	73
37	Live with tumor*.ab. or Live with tumor*.at.	31

38	Live with Neoplasm*.ab. or Live with Neoplasm*.at.	0
39	Live with Malignanc*.ab. or Live with Malignanc*.at.	0
40	Chronic Disease*.ab. or Chronic Disease*.at.	85443
41	Disease*, Chronic.ab. or Disease*, Chronic.at.	8727
42	Chronic Illness*.ab. or Chronic Illness*.at.	22210
43	Illness*, Chronic.ab. or Illness*, Chronic.at.	493
44	Chronic Condition*.ab. or Chronic Condition*.at.	27580
45	Condition*, Chronic.ab. or Condition*, Chronic.at.	1533
46	Live with Chronic Disease*.ab. or Live with Chronic Disease*.at.	23
47	Live with Chronic condition*.ab. or Live with Chronic condition*.at.	18
48	23 or 24 or 25 or 26 or 27 or 28 or 29 or 30 or 31 or 32 or 33 or 34 or 35 or 36 or 37 or 38 or 39 or 40 or 41 or 42 or 43 or 44 or 45 or 46 or 47	3439894
49	22 and 48	194103
50	transition*.ab. or transition*.at.	512088
51	health transition*.ab. or health transition*.at.	741
52	continuity of patient care.ab. or Continuity of Patient Care.at.	442
53	Care Continuity, Patient.ab. or Care Continuity, Patient.at.	7
54	Patient Care Continuity.ab. or Patient Care Continuity.at.	26
55	Continuum of Care.ab. or Continuum of Care.at.	3569
56	Care Continuum.ab. or Care Continuum.at.	2375
57	Continuity of Care.ab. or Continuity of Care.at.	9888
58	Care Continuity.ab. or Care Continuity.at.	757
59	aftercare.ab. or aftercare.at.	4656
60	after care.ab. or after care.at.	591752
61	after treatment*.ab. or after treatment*.at.	1518143
62	Follow-Up Care*.ab. or Follow-Up Care*.at.	7407
63	Care*, Follow-Up.ab. or Care*, Follow-Up.at.	8107
64	Hospital to Home Transition.ab. or Hospital to Home Transition.at.	99
65	Hospital to Home*.ab. or Hospital to Home*.at.	3782
66	Home, Hospital to.ab. or Home, Hospital to.at.	1028
67	Patient discharge*.ab. or Patient discharge*.at.	6367
68	Discharge, Patient.ab. or Discharge, Patient.at.	897
69	Discharge Planning*.ab. or Discharge Planning*.at.	5612
70	Planning*, Discharge.ab. or Planning*, Discharge.at.	187
71	Hando* to adult care.ab. or Hando* to adult care.at.	0
72	Hand O* to adult care.ab. or Hand O* to adult care.at.	0
73	Signo* to adult care.ab. or Signo* to adult care.at.	0
74	Sign O* to adult care.ab. or Sign O* to adult care.at.	0
75	Health transition*.ab. or Health transition*.at.	741
76	Healthcare transition*.ab. or Healthcare transition*.at.	222

77	Health care transition*.ab. or Health care transition*.at.	398
78	Nursing hando*.ab. or Nursing hando*.at.	254
79	Patient hando*.ab. or Patient hando*.at.	765
80	Hando*, Patient.ab. or Hando*, Patient.at.	97
81	Patient Hand O*.ab. or Patient Hand O*.at.	72
82	Hand O*, Patient.ab. or Hand O*, Patient.at.	73
83	Patient Sign O*.ab. or Patient Sign O*.at.	28
84	Sign O*, Patient.ab. or Sign O*, Patient.at.	30
85	Patient signo*.ab. or Patient signo*.at.	2
86	Signo*, Patient.ab. or Signo*, Patient.at.	2
87	Nursing Hando*.ab. or Nursing Hando*.at.	254
88	Hando*, Nursing.ab. or Hando*, Nursing.at.	17
89	Nursing Hand Over*.ab. or Nursing Hand Over*.at.	2
90	Nursing Hand Out*.ab. or Nursing Hand Out*.at.	0
91	Hand Out*, Nursing.ab. or Hand Out*, Nursing.at.	0
92	Hand Over*, Nursing.ab. or Hand Over*, Nursing.at.	0
93	Clinical hando*.ab. or Clinical hando*.at.	278
94	Hando*, Clinical.ab. or Hando*, Clinical.at.	28
95	Patient transfer*.ab. or Patient transfer*.at.	2721
96	Transfer* to adult care.ab. or Transfer* to adult care.at.	283
97	Transition* to adult*.ab. or Transition* to adult*.at.	3347
98	Transition* to adult care.ab. or Transition* to adult care.at.	590
99	Transfer*, Patient.ab. or Transfer*, Patient.at.	732
100	Patient Transition*.ab. or Patient Transition*.at.	473
101	Transition*, Patient.ab. or Transition*, Patient.at.	184
102	Care Transition*.ab. or Care Transition*.at.	2837
103	Transition*, Care.ab. or Transition*, Care.at.	3527
104	Transition of Care.ab. or Transition of Care.at.	1503
105	Transition of health Care.ab. or Transition of health Care.at.	114
106	Health Care Transition.ab. or Health Care Transition.at.	297
107	Care Transition*, Health.ab. or Care Transition*, Health.at.	6
108	Health Care Transitions.ab. or Health Care Transitions.at.	103
109	Transition*, Health Care.ab. or Transition*, Health Care.at.	104
110	Patient Turfing*.ab. or Patient Turfing*.at.	0
111	Turfing*, Patient.ab. or Turfing*, Patient.at.	0
112	Patient Dumping.ab. or Patient Dumping.at.	54
113	Dumping, Patient.ab. or Dumping, Patient.at.	2
114	Care Retention.ab. or Care Retention.at.	212
115	Transfer from Pediatric to Adult Care.ab. or Transfer from Pediatric to Adult Care.at.	51
116	Pediatric Transition To Adult Care.ab. or Pediatric Transition To Adult Care.at.	0
117	Transfer* to Adult Care.ab. or Transfer* to Adult Care.at.	283

118	Care*, Transitional.ab. or Care*, Transitional.at.	64
119	Transition* Care*.ab. or Transition* Care*.at.	3611
120	Home Transition*.ab. or Home Transition*.at.	267
121	Transition*, Home.ab. or Transition*, Home.at.	485
122	50 or 51 or 52 or 53 or 54 or 55 or 56 or 57 or 58 or 59 or 60 or 61 or 62 or 63 or 64 or 65 or 66 or 67 or 68 or 69 or 70 or 71 or 72 or 73 or 74 or 75 or 76 or 77 or 78 or 79 or 80 or 81 or 82 or 83 or 84 or 85 or 86 or 87 or 88 or 89 or 90 or 91 or 92 or 93 or 94 or 95 or 96 or 97 or 98 or 99 or 100 or 101 or 102 or 103 or 104 or 105 or 106 or 107 or 108 or 109 or 110 or 111 or 112 or 113 or 114 or 115 or 116 or 117 or 118 or 119 or 120 or 121	2494522
123	22 and 48 and 122	28675
124	transition readiness.ab. or transition readiness.at.	406
125	readiness.ab. or readiness.at.	24731
126	read*.ab. or read*.at.	662970
127	handiness.ab. or handiness.at.	81
128	Preparation.ab. or Preparation.at.	346728
129	Preparedness.ab. or Preparedness.at.	19411
130	Parar*.ab. or Parar*.at.	2800
131	124 or 125 or 126 or 127 or 128 or 129 or 130	1016116
132	122 and 131	92705
133	49 and 132	938
